# Hub microRNAs and genes in the development of atrial fibrillation identified by weighted gene co-expression network analysis

**DOI:** 10.1186/s12920-021-01124-5

**Published:** 2021-11-15

**Authors:** Qiang Qu, Jin-Yu Sun, Zhen-Ye Zhang, Yue Su, Shan-Shan Li, Feng Li, Ru-Xing Wang

**Affiliations:** 1 Department of Cardiology, The Affiliated Wuxi People’s Hospital of Nanjing Medical University, No. 299, Qingyang Road, Wuxi, 214023 China; 2 Department of Cardiology, The First Affiliated Hospital of Nanjing Medical University, Nanjing, 210029 China

**Keywords:** Atrial fibrillation, Weighted gene co-expression network analysis, Hub microRNAs, Hub genes, Inflammation

## Abstract

Co-expression network may contribute to better understanding molecular interaction patterns underlying cellular processes. To explore microRNAs (miRNAs) expression patterns correlated with AF, we performed weighted gene co-expression network analysis (WGCNA) based on the dataset GSE28954. Thereafter, we predicted target genes using experimentally verified databases (ENOCRI, miRTarBase, and Tarbase), and overlapped genes with differentially expressed genes (DEGs) from GSE79768 were identified as key genes. Integrated analysis of association between hub miRNAs and key genes was conducted to screen hub genes. In general, we identified 3 differentially expressed miRNAs (DEMs) and 320 DEGs, predominantly enriched in inflammation-related functional items. Two significant modules (red and blue) and hub miRNAs (hsa-miR-146b-5p and hsa-miR-378a-5p), which highly correlated with AF-related phenotype, were detected by WGCNA. By overlapping the DEGs and predicted target genes, 38 genes were screened out. Finally, 9 genes (i.e. *ATP13A3, BMP2, CXCL1, GABPA, LIF, MAP3K8, NPY1R, S100A12, SLC16A2*) located at the core region in the miRNA-gene interaction network were identified as hub genes. In conclusion, our study identified 2 hub miRNAs and 9 hub genes, which may improve the understanding of molecular mechanisms and help to reveal potential therapeutic targets against AF.

## Introduction

Atrial fibrillation (AF), the most common sustained cardiac arrhythmia, is expected to affect one quarter of middle-aged adults worldwide, primarily at the age of > 70 years [1]. According to the epidemiological investigation, the overall prevalence was estimated to be 1–4% in Australia, Europe and the USA [1, 2]. The number continued to increase as the population is ageing, reaching 4 per 1000 person-years in 2016 [3, 4]. AF, which accounts for the majority (≈ 70%) of tachyarrhythmia, places a substantial economic burden on healthcare systems [5].

AF has been associated with electrophysiological and structural remodeling, characterized by rapid and uncoordinated atrial activity [6]. The mechanisms underlying AF are complex and multifactorial, and are broadly classified as trigger and substrate, which lead to an increased risk of stroke, heart failure, and premature death [7]. Among them, several molecular factors such as abnormal Ca^2+^ handling, fibrosis, and inflammation, are known to be relevant to the development of AF at systemic level [8–11]; however, AF treatment remains challenging because its exact mechanisms at cellular/molecular level are not fully defined. Therefore, further basic investigation to examine predictive and prognostic biomarkers in AF management will be of great interest.

MicroRNAs (miRNAs) are short (19–25 nucleotides) non-coding RNAs that regulate gene expression by binding to complementary sequences of mRNAs [12, 13]. One miRNA may target hundreds of different mRNAs, while one mRNA may be regulated by multiple miRNAs [14]. The miRNA-gene interaction network can be used to uncover potential biomarkers for disease detection and treatment. A large number of studies have demonstrated the important role of miRNAs in regulating cardiac excitability and arrhythmogenesis in various cardiovascular diseases [15–18]. However, data regarding regulating effects of miRNAs on the transition from sinus rhythm to AF, including potential mechanisms, are limited.

Weighted gene co-expression network analysis (WGCNA) is a bioinformatics algorithm to detect causal relationship between genome and clinical features based on alterations in transcriptome expression patterns under pathological conditions [19]. Co-expression network, composing various biological processes, is grouped into several modules to explore the association between genes and clinical features [20]; meanwhile, genes in the same module are usually functionally-linked and have similar regulating effects on signal pathways [21]. The most central genes tend to play a vital role within a specific module and are generally regarded as hub genes. WGCNA, unlike differential expression analysis, has been confirmed as a powerful systematic analysis method to recognize higher-order association across genes rather than detect disease-related individual genes. This study aimed to investigate hub miRNAs and genes in patients with AF by combining differential expression analysis and WGCNA.

## Materials and methods

### Data sources

**Fig. 1:**
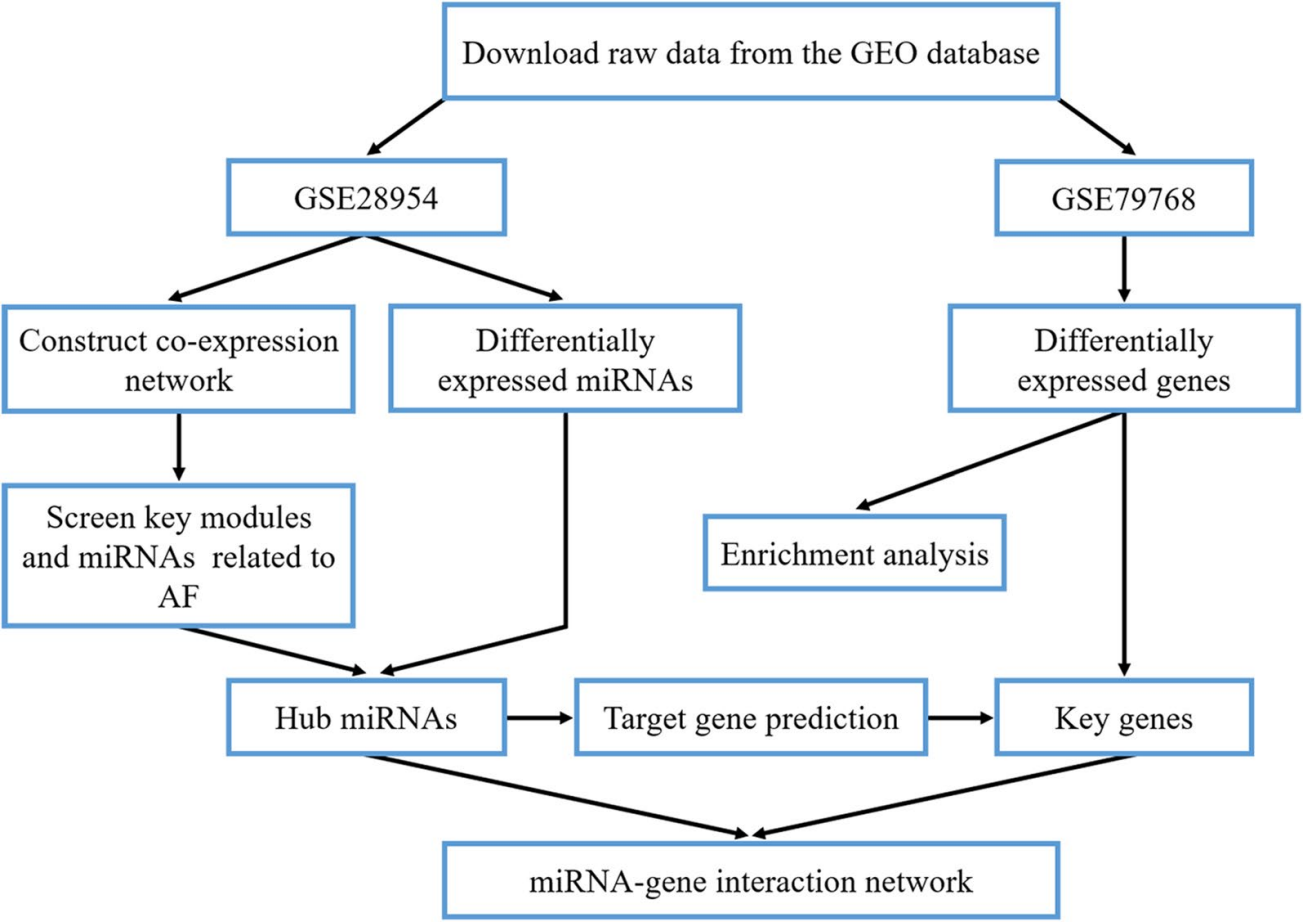
Flow chart of data preparation, processing, analysis, and validation. AF, atrial fibrillation; GEO, gene expression omnibus; miRNA, microRNA

The workflow of this study is shown in Fig. 1. Microarray-based miRNA and mRNA expression data were obtained from the Gene Expression Omnibus (GEO) database if they met the following selection criteria [22]: (1) expression data were from atrial tissue samples in non-AF and AF patients; (2) study type was restricted to expression profiling by array; (3) organism was restricted to Homo sapiens; and (4) raw/processed expression data were public and available. The expression profiles of GSE28954 and GSE79768, separately containing miRNA and gene expression data, were included to screen hub miRNAs and genes related to the development of AF [23, 24].

### Probe reannotation and data preprocessing

The expression profiles were acquired by reannotating probes in the datasets, as previously described [25]. The probe set was mapped to RefSeq transcript ID based on the latest version of the annotation file. The average expression value was used if multiple probes were applied to detect one miRNA or mRNA. Then, we performed background correction, log_2_-transformation, and quantile normalization using the ‘normalize Between Arrays’ function of the ‘linear models for microarray data (limma)’ package in R software v3.6.3 [26, 27]. No missing expression value was detected during data processing.

### Identification of differentially expressed miRNAs (DEMs) and genes (DEGs)

We used the ‘limma’ package to screen the DEMs and DEGs between normal rhythm and AF samples with a threshold of false discovery rate (FDR) < 0.05 and |log_2_ fold-change (FC)| > 0.5. Thereafter, we visualized the DEMs and DEGs as a volcano plot and a heatmap using the ‘ggplot2’ and ‘pheatmap’ package, respectively.

### Functional enrichment analysis of the DEGs

To identify characteristic biological and biochemistry pathways in the development of AF, we used the ‘clusterProfiler’ package [28], together with Metascape [29], to perform gene ontology (GO) [30, 31] and Kyoto Encyclopedia of Genes and Genomes (KEGG) pathway enrichment analysis [32–34] for the DEGs, with cut-offs of *q *value < 0.05 and *q *value < 0.20 established for significant biological processes and biological processes, respectively. The q-value of a test measures the proportion of false positives incurred (FDR) control when that particular test is called significant [35].

### Weighted gene co-expression network construction and module detection

To explore the relationship between genome and clinical features, we used the ‘WGCNA’ package to construct a co-expression network based on the DEMs. After assessing the presence of obvious outliers by sample clustering, we used the ‘one-step network construction’ function to construct the miRNA co-expression network. We used the ‘pick-Soft-Threshold’ function to calculate the soft-thresholding power β and converted the adjacency into a topological overlap matrix. Then, we conducted average linkage hierarchical clustering and dynamic tree cut at a merging threshold of 0.25. The co-expression network was visualized as a heatmap based on topological overlap dissimilarity and the cluster dendrogram.

### Screening key modules

We used module eigengene (ME), the first principal component of module expression, to represent the expression profile of module miRNAs [36]. To validate module-trait relationships (MTRs, defined as the correlation between MEs and clinical features) of miRNA modules, we categorized miRNAs into corresponding modules according to the constructed modules [37]. We calculated the ME of each module and included related clinical features. Then, we calculated gene significance (GS, defined as the log_10_-transformation of *P*-value in the linear regression slope between gene expression and clinical features) and module significance (MS, described as the average GS of all genes in the module) to further assess correlation intensity between a miRNA module and a clinical feature [25, 38]. In general, a module with the highest MS value was regarded as the key module for further analysis.

### Hub miRNA identification and key gene validation

After overlapping miRNAs from the key module with the DEMs from the dataset GSE28954, all of which were regarded as key miRNAs, we further calculate module membership (MM, representing the association between expression and ME) and GS (representing the association between expression and clinical features) to identify hub miRNAs. In general, key miRNAs with high values of MM and GS were regarded as hub miRNAs. The hub miRNAs were uploaded to 3 experimentally verified miRNA target prediction databases (ENOCRI [39, 40], miRTarBase [41], Tarbase [42]) to map corresponding target genes, predict the interaction between miRNAs and genes, and construct a miRNA-target gene regulatory network. Further, we overlapped predicted target genes and the DEGs to determine key genes.

### Construction of the miRNA-gene interaction network

To reveal the principle of cellular organization and predict protein functions, we constructed a protein–protein interaction (PPI) network of the DEGs by Metascape, an online tool for analyzing system-level datasets. Then, we constructed a miRNA-gene interaction network based on upstream and downstream relationship between hub miRNAs and key genes. After analyzing the interaction between key genes using the STRING database v11.0 with a threshold of confidence score > 0.150, we illustrated the network graph and merged the key gene interaction network and miRNA-target gene regulatory network into a miRNA-gene interaction network by Cytoscape v3.6.2 [43]. However, the STRING database, which contains both positive and negative regulatory relationship between miRNAs and genes, does not provide their causal association. The core region with a dense connection in the network was detected by the ‘cytoHubba’ plugin via mixed character calculation.

## Results

### Microarray data normalization and identification of the DEMs and DEGs

**Fig. 2:**
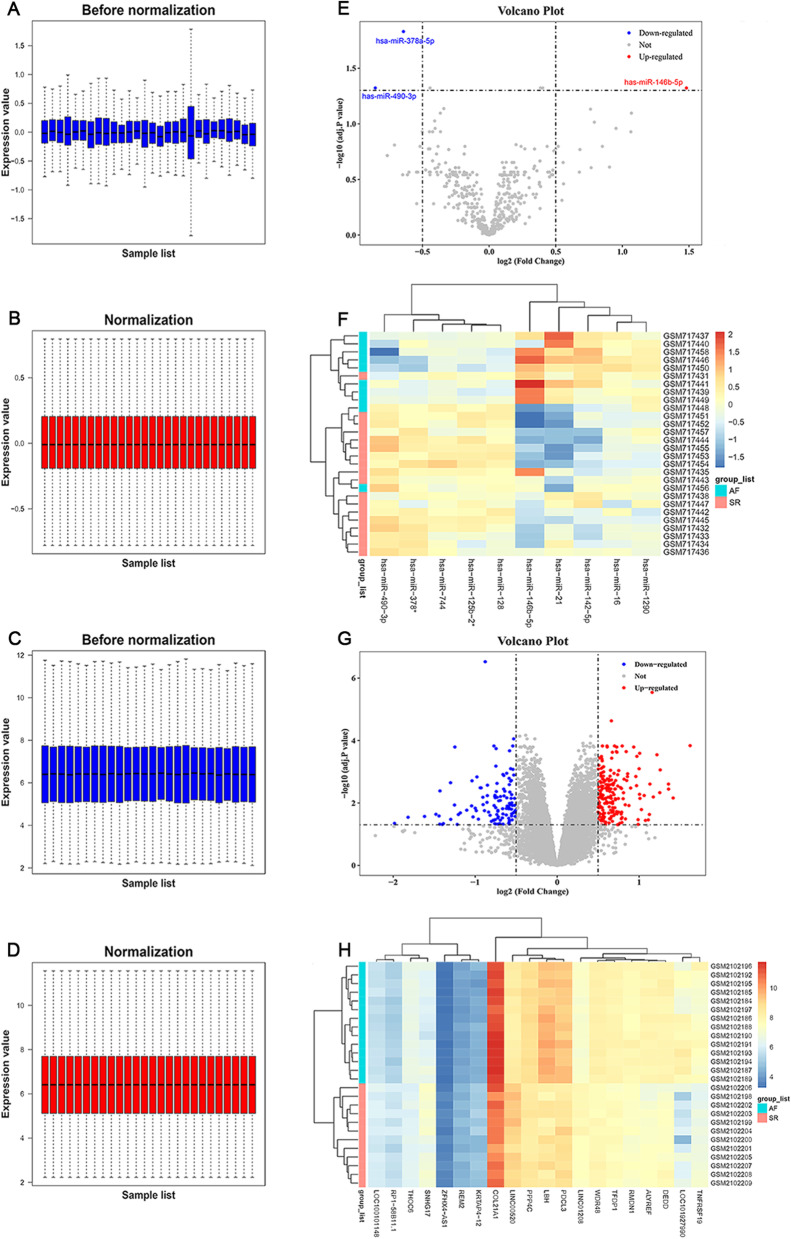
The normalization of microarray expression profiles and visualization of differentially expressed miRNAs and genes between normal rhythm and AF samples. **A**, **B** The normalization of the dataset GSE28954. **C**, **D** The normalization of the dataset GSE79768. **E**, **F** The volcano plot and heatmap to show differential miRNA expression from GSE28954 with a threshold of false discovery rate (FDR) < 0.05 and |log_2_ fold-change (FC)| > 0.5. **G**, **H** The volcano plot and heatmap to show differential gene expression from GSE79768 with a threshold of FDR < 0.05 and |log_2_ FC| > 0.5. AF, atrial fibrillation; miRNA, microRNA

In the dataset GSE28954, 10 AF and 18 non-AF samples were included, with no further information given; in the dataset GSE79768, 14 AF and 12 non-AF samples were included with a mean age of 55.4 years, comprising 10 (38.5%) men and 16 (61.5%) women. After data preprocessing of AF chip expression datasets GSE28954 and GSE79768 (Fig. 2A–D), 3 DEMs and 320 DEGs were identified at cut-off levels of FDR < 0.05 and |log_2_ FC| > 0.5. Among the 3 DEMs, hsa-miR-146b-5p was up-regulated in atrial tissue samples from AF patients, whereas hsa-miR-378a-5p and hsa-miR-490-3p were down-regulated; among the 320 DEGs, 203 DEGs were up-regulated in atrial tissue samples from AF patients, whereas 117 DEGs were down-regulated. The volcano plot and heatmap are shown in Fig. 2E–H.

### Functional enrichment analysis of the DEGs

**Fig. 3:**
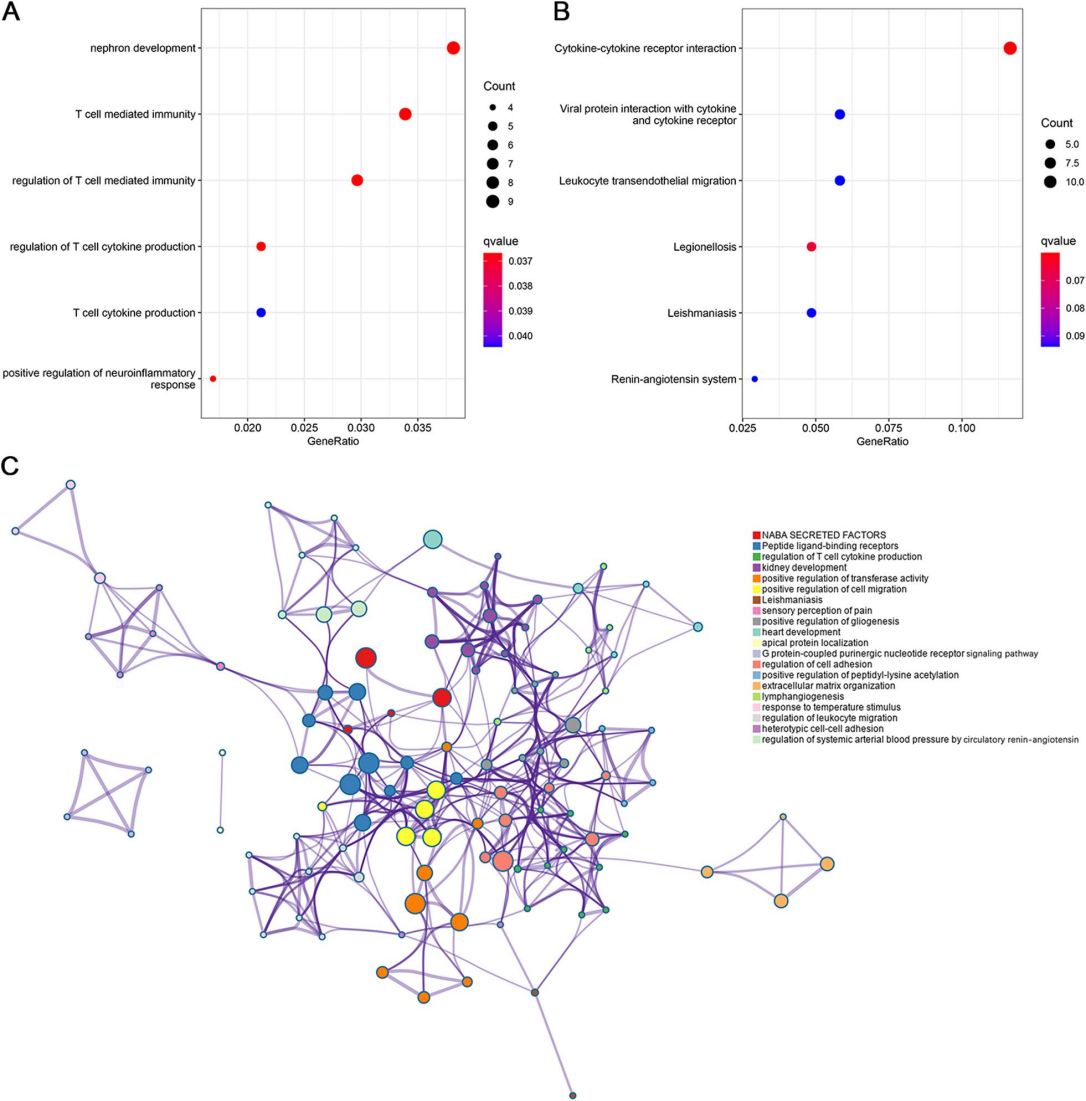
**A** Gene ontology and **B** Kyoto Encyclopedia of Genes and Genomes pathway enrichment analysis for the DEGs. The dot color reflects the statistical significance, whereas the dot size reflects the number of the DEGs in corresponding pathways. **C** The network of enriched terms. Each node that represents an enriched item is colored by cluster ID; nodes with the same cluster ID are typically close to each other. DEG, differentially expressed gene

We performed functional enrichment analysis of the DEGs based on the GO and KEGG databases. As shown in Fig. 3A, the enriched biological processes were mainly involved in nephron development, T cell mediated immunity, regulation of T cell mediated immunity, regulation of T cell cytokine production, T cell cytokine production, and positive regulation of neuroinflammatory response. Moreover, the KEGG enrichment analysis showed that cytokine-cytokine receptor interaction was the most enriched pathway, followed by neuroactive ligand-receptor interaction, viral protein interaction with cytokine and cytokine receptor, and chemokine signaling pathway (Fig. 3B).

Figure 3C summarizes the top 20 clusters of pathway and process enrichment analysis in Metascape, of which 5 representative enrichment terms were closely associated with inflammation, including regulation of T cell cytokine production, positive regulation of cell migration, regulation of cell adhesion, regulation of leukocyte migration, and heterotypic cell–cell adhesion. In general, functional enrichment analysis of the DEGs supported the involvement of inflammation in the development of AF.

### Construction of the co-expression network

**Fig. 4:**
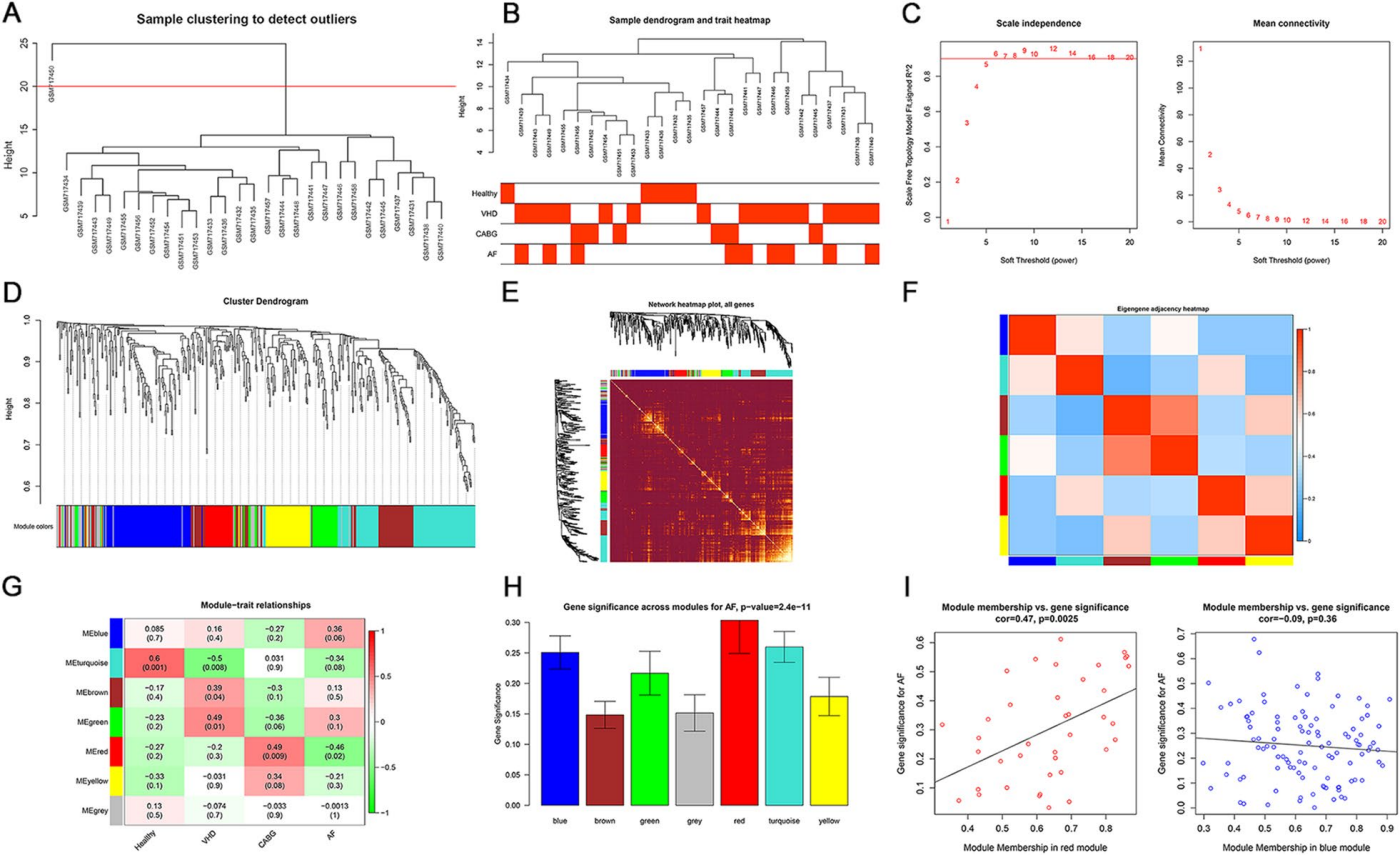
The identification of key modules by weighted gene co-expression network analysis. **A** Clustering dendrogram of samples in the dataset GSE28954. The red line in the dendrogram is the cut line for outlier detection, with samples cut by this line considered as outliers. **B** Clustering dendrogram of 9 AF and 18 non-AF samples. **C** Analysis of network topology for various soft-thresholding powers. **D** Clustering dendrogram of miRNAs, with dissimilarity based on topological overlap, together with assigned module colors. **E** The visualization of the co-expression modules using a heatmap. The degree of overlap increases with increasing intensity of yellow. **F** Eigengene adjacency heatmap of different co-expression modules. **G** The heatmap to show the correlation between module eigengenes and overall health status (healthy, valvular heart disease, coronary artery bypass graft, and AF). **H** The distribution of average expression significance across modules related to AF. **I** The scatter plot of module eigengenes in red module and blue module. AF, atrial fibrillation; miRNA, microRNA

We used average linkage method, together with Pearson’s correlation method, to cluster the samples of GSE28954; meanwhile, we used distance across samples in Pearson’s correlation matrices to assess quality of microarray and locate obvious outliers. The samples were included if they met the inclusion criteria: data cut-off height of < 20 (Fig. 4A, B). The soft-thresholding power was set at 5 since scale-free topology index reached 0.9 and mean connectivity was relatively high (Fig. 4C). In general, 7 modules were identified by average linkage hierarchical clustering, with none merged due to dissimilarity (data cut-off height of < 0.25). Turquoise module was the largest module with 123 miRNAs, followed by blue module with 106 miRNAs; conversely, red module was the smallest module with 39 miRNAs. Grey module, which included miRNAs belonging to no other module, was were dismissed in the following analysis. The cluster dendrogram of the DEMs is shown in Fig. 4D. Moreover, we analyzed the interaction association between modules. The network heatmap, in combination with eigengene adjacency heatmap of miRNAs, demonstrated a high level of independence across co-expression clusters (Fig. 4E, F).

### Constitution of module-trait relationship and detection of key modules

To identify miRNAs related to a specific clinical feature, we used correlation analysis to explore the association of modules with overall health status (healthy, valvular heart disease, coronary artery bypass graft, and AF). Red module had the highest negative correlation with the development of AF (r = − 0.46, *P* = 0.02), whereas blue module had the highest positive correlation (r = 0.36, *P* = 0.06; Fig. 4G). Moreover, Fig. 4H shows that they had the highest MS values across all modules. Therefore, red module and blue module were regarded as key modules for AF. Significant correlation between MM and GS were also seen for the development of AF (Fig. 4I).

### Identification of hub miRNAs

After overlapping miRNAs in key modules with the DEMs from the dataset GSE28954, hsa-miR-146b-5p and hsa-miR-378a-5p were regarded as the 2 key miRNAs. Furthermore, the |MM| and |GS| values of hsa-miR-146b-5p were 0.48 and 0.62, respectively, whereas the numbers separately reached 0.46 and 0.68 in hsa-miR-378a-5p. Therefore, hsa-miR-146b-5p and hsa-miR-378a-5p were further regarded as hub miRNAs.

### Target gene prediction and key gene validation

A total of 2935 target genes and 3000 miRNA-gene pairs were obtained after miRNA-gene mapping by electronic databases (i.e. ENOCRI, miRTarBase, Tarbase). The association between AF-related hub miRNAs and genes was further verified by overlapping target genes and the DEGs. Finally, 38 genes were regarded as key genes.

### Analysis of the miRNA-gene interaction network

**Fig. 5:**
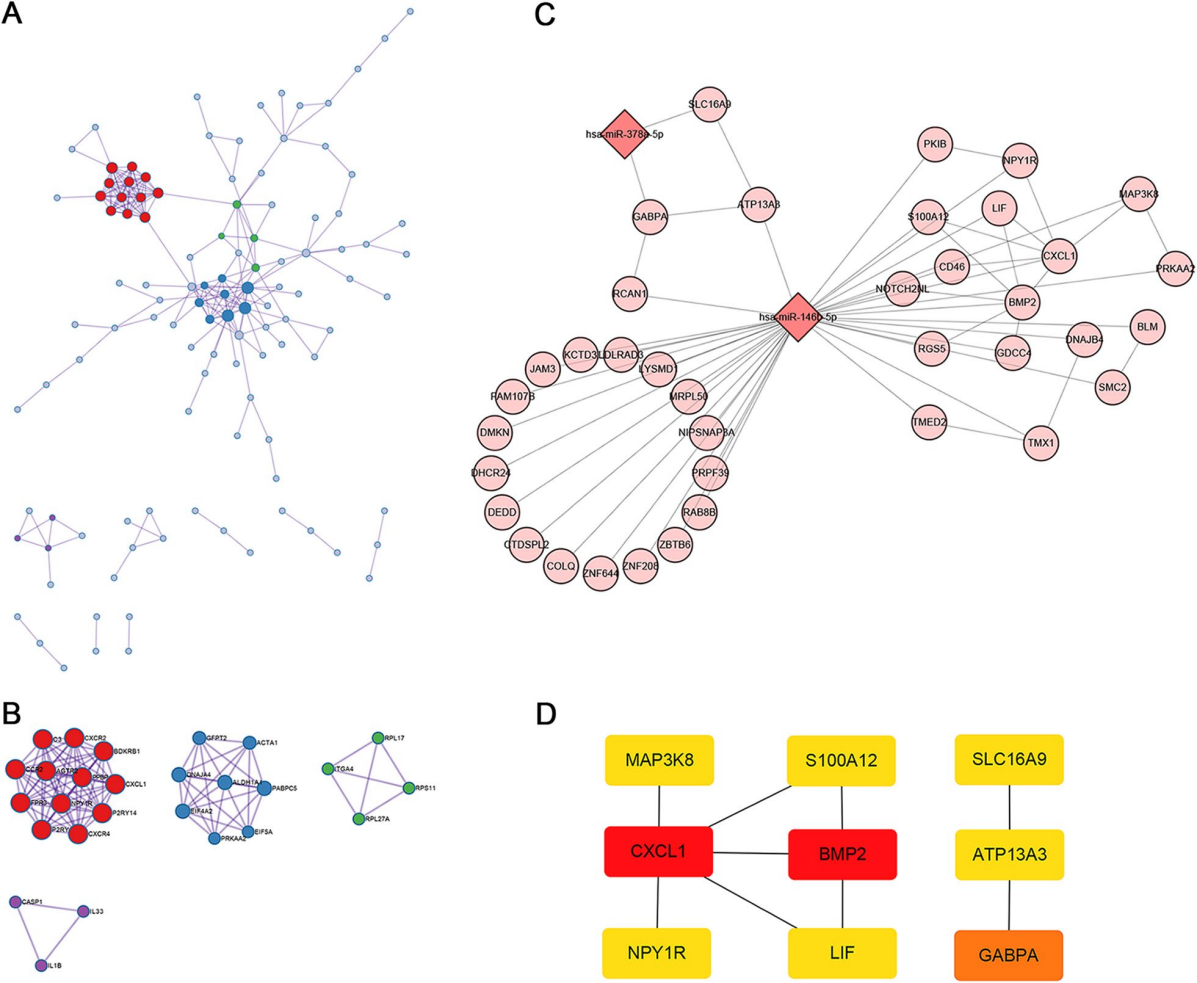
**A** The protein–protein interaction network of differentially expressed genes. The network contains the subset of proteins that form physical interactions with at least one other member. **B** Four molecular complex detection components identified by pathway and process enrichment analysis. **C** The construction of the miRNA-gene interaction network. The hub miRNAs are shown in dark pink, whereas the key genes are shown in light pink. The edge between two nodes represents the interaction between miRNAs and/or genes. **D** Nine hub genes identified by the ‘cytoHubba’ plugin via mixed character calculation. The significance of hub genes increases with increasing intensity of the dot color. miRNA, microRNA

To better understand the interaction between the DEGs, we used Metascape to perform PPI enrichment analysis and illustrate a list of important gene components (Fig. 5A). Then, we performed pathway and process enrichment analysis and identified 4 molecular complex detection (MCODE) components (Fig. 5B). The results showed that biological functions of MCODE components were mainly involved in G protein-coupled receptor activity, generation of precursor metabolites and energy, protein translation process, and positive regulation of cytokine secretion.

Moreover, we constructed a miRNA-gene interaction network to determine hub genes related to the regulation of key signaling pathways in the development of AF. As shown in Fig. 5C, 40 nodes (2 miRNAs and 38 genes) and 57 pairs of interaction relationship were observed in the network. The hsa-miR-146b-5p was the top hub node in the network with a degree of 36. Further, the ‘cytoHubba’ plugin detected 9 hub genes based on topological parameters of the network, including *ATP13A3, BMP2, CXCL1, GABPA, LIF, MAP3K8, NPY1R, S100A12,* and *SLC16A2* (Fig. 5D).

## Discussion

AF is the most common sustained cardiac arrhythmias, characterized by rapid and uncoordinated atrial activity, which leads to an increased risk of complications (e.g. stroke, heart failure, premature death) and constitutes a substantial economic burden worldwide. However, the mechanisms underlying AF are intricate and have not been fully understood; there remains an unmet medical need in the treatment of AF to date. In the present study, we used miRNA and gene expression data to identify predictive and prognostic biomarkers in AF management. Furthermore, we performed integrated analysis to construct a miRNA-gene interaction network for further validation.

Our study performed WGCNA to identify miRNA co-expression modules and the MTRs related to AF; the results showed a significant correlation of red module and blue module with the development of AF. The differential expression analysis showed that 3 miRNAs and 38 key genes were differentially expressed in atrial tissue samples from AF patients. Overall, we identified 2 hub miRNAs (hsa-miR-146b-5p and hsa-miR-378a-5) and constructed a miRNA-gene interaction network containing 9 hub genes (*ATP13A3, BMP2, CXCL1, GABPA, LIF, MAP3K8, NPY1R, S100A12,* and *SLC16A2*), of which 7 (*ATP13A3, BMP2, CXCL1, LIF, MAP3K8, NPY1R,* and *S100A12*) were linked to hsa-miR-146b-5p. Functional enrichment analysis, including biological functions of MCODE components, showed that the DEGs were mainly involved in inflammation-related items, highlighting a central role of inflammation in the development of AF.

New-onset or exacerbation of AF results from the interaction between trigger and substrate conceptually [6]; moreover, the maintenance of AF requires a vulnerable substrate, largely attributable to genetic predisposition, cardiovascular neurohormonal regulation, and disease-related cardiac remodeling [44, 45]. Cardiac electrophysiology remodeling represents the change in properties of ion channels, whereas structural remodeling represents the change in organization structure (including atrial dilatation and fibrosis) [46]. While the exact mechanism underlying the development of AF has not been clearly established, atrial dilatation and fibrosis are known to influence a variety of biological processes, primarily through regulation of angiotensin II and related mediators (e.g. transforming growth factor [TGF]-β1, platelet derived growth factor [PDFG], connective tissue growth factor [CTGF]) [47]. AF can stimulate the release of pro-inflammatory cytokines and chemokines related to cardiovascular disease and tissue injuries such as angiotensin II, interleukin (IL)-6, IL-8, and tumor necrosis factor (TNF)-α [48]. In addition, accumulating evidence has demonstrated that mechanical stretch and injuries stimulate leukocyte activation and the release of pro-inflammatory mediators, including nicotinamide adenine dinucleotide phosphate oxidase-derived reactive oxygen species, fibroblast growth factors, and regulatory hormones [49].

Inflammation has been associated with a clinically relevant effect on the increase of cardiac/serum inflammatory biomarkers, and was shown to regulate expression patterns of specific miRNAs and genes in AF patients, thereby potentiating the fibrosis of an established AF substrate [50–53]. AF, largely driven by inflammation, also has been reported to initiate additional inflammatory responses, aggravate atrial remodeling, and deteriorate cardiac function [54]. Such interactions may constitute a key inducement of the transformation from fibroblasts into myofibroblasts and attain significance for the mechanism underlying AF. Other inflammation-related factors such as oxidative stress, apoptosis, and thrombogenesis from the bioactivation of coagulation cascade, were also generally associated with the development of AF [50]. Inflammation, particularly those related to atrial fibrosis, represents an important mechanism of AF. Herein, we summarize a list of miRNAs and genes potentially associated with the inflammatory process in the presence of AF.

BMPs, bone morphogenetic proteins, constitute the largest branch of TGF-β family ligands. In addition to enhancing bone regeneration, BMPs also exert anti-inflammatory activity by inhibiting inflammatory cell infiltration in patients with cardiovascular disease [55, 56]. BMP2 and BMP4 have been reported to participate in the development of cardiac function, and so their interruption or discontinuation can lead to cardiac function abnormalities in adulthood [57, 58]. Howden et al*.* [59] demonstrated that QRS duration and ST interval were significantly reduced in BMP2/BMP4 knock-out mice compared with wide-type group, suggesting a potential role for BMP2 and BMP4 in the development of short QT interval syndrome and following initiation of AF. BMP7, an inhibitor of apoptosis, fibrosis, and calcification, has been shown to stimulate the differentiation from pro-inflammatory infiltrated monocytes to anti-inflammatory M2 macrophages and reduce the progression of cardiac dysfunction in multiple cardiovascular diseases [60]. Our results indicated that BMPs were down-regulated in subjects with AF than in subjects with sinus rhythm, with its expression significantly correlating with the presence of AF.

CXCL1, C-X-C motif chemokine ligand 1, is a member of the alpha chemokine subfamily involved in enhanced neutrophil chemotaxis and phagocytosis during inflammation. It has been suggested that the level of CXCL1 was in keep with the extent of myocardial inflammation and the percentage of CD14^++^DN16^−^ monocytes [61]. Moreover, CXCL1-induced myocardial or systemic inflammation in patients with acute cardiomyopathy was significantly associated with monocyte adhesion, macrophage infiltration into the myocardium, and the release of inflammatory cells from bone marrow. Fan et al*.* [62] also demonstrated that the level of CXCL1 was significantly higher in the AF group than in the control group (by stimulation of cytokines), which was consistent with that seen in our study. Overall, molecular mechanisms of action of CXCL1 behind AF have not been fully elucidated, and remain to be confirmed.

LIF, leukemia inhibitory factor, is a secreted glycoprotein of interleukin-6-type cytokine family. LIF has been shown to transmit biological information through a heterodimer receptor complex comprising LIF receptor and Interleukin 6 signal transducer in a variety of inflammatory processes, including acute phase reaction, hematopoiesis, bone metabolism, and cancer progression [63]. Our study indicated that LIF was down-expressed in atrial tissue of AF patients, suggesting that inflammation-related atrial fibrosis was of particular importance for the development of AF. However, an earlier animal study in adult pigs found no significant difference between the AF group and the control group, which was conflicting with that seen in previous reports [64]. Additional studies would help to determine the position of LIF in the development of AF.

Consistent with the results of recent reports, our study further demonstrated that hsa-miR-146b-5p was over-expressed in AF patients [53, 65]. has-miR-146b-5p has been reported to function as an intracellular mediator in maladaptive remodeling of atrial fibrosis by breaking the balance of MMP/TIMP axis and increasing the level of collagen content of cardiac fibroblasts in AF patients [53, 66]. Emerging evidence suggests that vascular smooth muscle cells (VSMCs) can achieve perceived plasticity and exert non-professional phagocytic activity to maintain inflammatory or senescent condition [67]. Notably, MBNL1 was significantly down-regulated via its interaction with hsa-miR-146b-5p, which was expected to influence VSMC proliferation and differentiation [68, 69]. In human monocytes and murine cardiomyocytes, has-miR-146b-5p regulated the expression of several pro-inflammatory mediators, including tumor necrosis factor-associated factor 6 (TRAF6), Interleukin-1 receptor (IL-1R)-associated kinase (IRAK1), IL-6, and signal transducer and activator of transcription 3 (STAT3) [70, 71]. Studies in mouse models showed that has-miR-146b-5p down-regulated TRAF6 expression (known to act upstream of the NF-κB pathway) under hypoxia conditions, thereby inhibiting cardiac fibrosis and preventing cardiac dysfunction in patients with heart failure [71]. In addition to hub genes described above, *ATP13A3*, *MAP3K8*, *NPY1R*, and *S100A12* were regulated by hsa-miR-146b-5p; their exact mechanisms of action underlying AF remain elusive, and therefore, further molecular biology studies are warranted in the future. In general, hsa-miR-146b-5p may be a central mediator in the development of AF.

hsa-miR-378a-5p was another hub miRNA that could influence the development of AF, primarily through reversible inhibition of *GABPA* and *SLC16A2*. Our results showed that they were down-regulated, in contrast to hsa-miR-378a-5p that was over-expressed in the presence of AF. hsa-miR-146b-5p, targeting anti-proliferative protein TOB2, has been shown to positively regulate the cell cycle and the following angiogenic process, thereby inducing or enhancing the development of AF by inflammation or fibrosis [72–74]. Despite the lack of studies on the association of GABPA with AF, GABPA has been reported to down-regulate genes involved in the inflammatory response and oxidative stress at transcriptional level [75]. Additional research is needed to fully elucidate the potential cardioprotective effects, if any, of SLC16A2 in the development of AF.

The main strength of this study is the use of atrial tissue samples from AF patients instead of commonly reported murine aneurysmal model or human VSMCs in miRNA studies, whose results need additional testing and analysis. Another strength is to provide an overview and perspective from the interaction between miRNAs and genes for AF management, despite requiring more in-depth investigation and further validation.

Our study has several limitations. First, although 2 independent datasets were used for integrated analysis, input data were insufficient to accurately identify and validate predictive and prognostic biomarkers in AF management. Second, given that a substantial proportion of AF samples from the dataset GSE28954 were strongly correlated with dilated atria due to valvular heart disease, our findings were expected to be outmost applicable to patients with AF and concurrent valvular heart disease, with a controversial generalizability to the whole population. Third, our study merely focused on detecting hub miRNAs and genes related to the development of AF, without considering epigenetic mechanisms behind multifactorial nature of disease, which are generally stable and are often affected by environmental factors. Fourth, despite advances in the pathogenesis of AF over the past decade, there remains a paucity of functional studies on hub molecules identified in our study, making it difficult to understand their implication for potential mechanisms of AF. Fifth, the paucity of confirmatory experiments is another noteworthy limitation; further results from molecular biology studies would be beneficial.

## Conclusion

WGCNA, in combination with a miRNA-gene interaction network, identified 2 hub miRNAs and 9 hub genes in the occurrence and progression of AF. This study may improve the understanding of molecular mechanisms and reveal potential therapeutic targets against AF. Further confirmatory experiments are warranted to validate these findings and elucidate underlying mechanisms.

## Data Availability

All expression data were acquired from the Gene Expression Omnibus (GEO, http://www.ncbi.nlm.nih.gov/geo/) database with the accession numbers GSE28954 and GSE79768.

## Abbreviations

AF: Atrial fibrillation
WGCNA: Weighted gene co-expression network analysis
DEM: Differentially expressed miRNA
DEG: Differentially expressed gene
FDR: False discovery rate
GO: Gene ontology
GS: Gene significance
KEGG: Kyoto encyclopedia of genes and genomes
MCODE: Molecular complex detection
ME: Module eigengene
MM: Module membership
MS: Module significance
MTR: Module-trait relationship
PPI: Protein–protein interaction
